# Repeated measures of body mass index and waist circumference in the assessment of mortality risk in patients with myocardial infarction

**DOI:** 10.1080/03009734.2018.1494644

**Published:** 2018-09-26

**Authors:** Lars Berglund, Ulf Risérus, Kristina Hambraeus

**Affiliations:** a Uppsala Clinical Research Center, Uppsala University, Uppsala, Sweden;; b Department of Public Health and Caring Sciences/Geriatrics, Uppsala University, Uppsala, Sweden;; c Department of Public Health and Caring Sciences, Uppsala University, Clinical Nutrition and Metabolism, Uppsala, Sweden;; d Department of Cardiology, Falun Hospital, Falun, Sweden;; e Department of Medical Sciences, Uppsala University, Uppsala, Sweden

**Keywords:** Body mass index, myocardial infarction, obesity paradox, repeated measurements, waist circumference

## Abstract

**Aims:** Weight loss is recommended for myocardial infarction (MI) patients with overweight or obesity. It has, however, been suggested that obese patients have better prognosis than normal-weight patients have, but also that central obesity is harmful. The aim of this study was to examine associations between repeated measures of body mass index (BMI) and waist circumference (WC), and all-cause mortality.

**Methods and results:** A total of 14,224 MI patients aged <75 years in Sweden between the years 2004 and 2013 had measurements of risk factors at hospital discharge. The patients’ BMI and WC were recorded in secondary prevention clinics two months and one year after hospital discharge. We collected mortality data up to 8.3 years after the last visit. There were 721 deaths. We used anthropometric measures at the two-month visit and the change from the two-month to the one-year visit. With adjustments for risk factors and the other anthropometric measure the hazard ratio (HR) per standard deviation in a Cox proportional hazard regression model for mortality was 0.64 (95% confidence interval [CI] 0.56–0.74) for BMI and 1.55 (95% CI 1.34–1.79) for WC, and 1.43 (95% CI 1.17–1.74) for a BMI decrease from month two to one year of more than 0.6 kg/m^2^. Low BMI and high WC were associated with the highest mortality.

**Conclusion:** High WC is harmful regardless of BMI in MI patients. Reduced BMI during the first year after MI is, however, associated with higher mortality, potentially being an indicator of deteriorated health.

## Introduction

In the year 2016 a total of 25,700 individuals in Sweden were affected by a myocardial infarction (MI). The proportion that died within 28 days after the event was 25%. Cardiovascular diseases continue to be the most common cause of death among both men and women (1). For MI patients several pharmacological and lifestyle interventions are recommended at hospital discharge in order to reduce the risk of early mortality.

Overweight and obesity are conditions associated with increased cardiovascular and non-cardiovascular mortality risks (2). Overweight and obese MI patients are generally recommended to decrease their body weight (3). This recommendation is, however, supported by very weak evidence, and the most recent guidelines on cardiovascular disease prevention from the European Society of Cardiology recognizes that the optimal level of body mass index (BMI) after a cardiovascular event still is one of the important remaining gaps in evidence (4).

Several observational studies show an inverse association between BMI and mortality in subjects with coronary artery disease (CAD). This phenomenon has been called the ‘obesity paradox’ (5–7). Waist circumference (WC), a measure of central obesity, may be a more valid measure of fat distribution than BMI in CAD patients, although WC carries larger measurement errors than BMI (8). There are only few studies examining the effect of central obesity on mortality in patients with CAD (9,10). Coutinho et al. conclude from a meta-analysis of four studies with 16,000 patients that central obesity is directly associated with higher mortality in individuals with CAD, whereas the opposite is valid for BMI (10). The direct effect of central obesity on mortality was observed even in subjects with normal BMI.

These studies use a single measurement of anthropometry (at hospital discharge) for each patient and are thus not able to consider fluctuations of BMI and WC. A single measurement of a predictor may not be sufficient to reveal its impact on an outcome, as post-MI changes in BMI and WC may also influence the risk, and it provides no basis for the recommendation for overweight and obese people to aim for a weight change after a myocardial infarction.

We examined how mortality, up to 8.3 years after a secondary prevention visit at one year after the infarction, was related to levels and changes of BMI and WC during the first year after hospital discharge.

## Material and methods

### Patient population

In this prospective cohort study, patients in Sweden with MI between the years 2004 and 2013 were treated at hospital, and their data, including BMI, were collected in the Swedish Web-system for Enhancement and Development of Evidence-based care in Heart disease Evaluated According to Recommended Therapies (SWEDEHEART) registry (11). After the hospital stay the patients returned at two months and one year after hospital discharge to secondary prevention clinics, and BMI and WC data from these visits were entered into the SWEDEHEART sub-register for Secondary Prevention after IHCU care (SEPHIA) (1). Dates for death, up to 8.3 years after the one-year visit, were collected from the Swedish National Population Registry.

Inclusion criteria to the analysis sample were that the patient was younger than 75 years at hospital discharge (since the SEPHIA registry had an upper age limit of 75), and was alive one year after hospital discharge. A further criterion was that the patient had complete data on variables from the hospital stay and had available anthropometric data from the two-month and one-year visits.

The study was approved by the local ethics committee (dnr EPN 2014/152).

### Anthropometric measures

Well-trained nurses or physicians measured anthropometric indices, with patients wearing only minimal clothing with no footwear during measurements. Weight and height were measured, with the patient standing, to the nearest 0.1 kg and 1 cm, respectively. If measurements were not possible, self-reported data were registered if the patient was able to communicate those. If not, data were registered as missing. BMI was calculated in the standard way: weight (kg) divided by square of height (m). WC was measured to the nearest cm with the patient standing, at the level midway between the lower rib margin and the iliac crest. The patient was asked to breathe out gently and relax during the measurement.

### Statistics

Continuous variables were described by means and standard deviations, and categorical variables were described by numbers and percentages.

In order to examine linearity of the associations between anthropometric variables and mortality we fitted and visualized a generalized additive model (GAM) (12) with a binomial link function. In the GAM model the anthropometric variables were adjusted for each other.

In Cox proportional hazards regression models we examined how all-cause mortality after the one-year visit was related to levels and changes of BMI and WC. The models were estimated with adjustments for the other anthropometric variable and covariates age and gender and further with adjustments for the other anthropometric variable and covariates age, gender, smoking, serum creatinine, diabetes, hypertension, hyperlipidaemia, previous myocardial infarction, previous stroke, previous congestive heart failure, diagnosis (STEMI/NSTEMI), percutaneous coronary intervention, coronary artery bypass grafting, and year of hospital discharge (2004–2013). Covariates were registered at the hospital stay at the time of the index MI.

Estimated models were presented with hazard ratios (HR) with 95% confidence intervals per standard deviation of BMI and WC levels, and *P* values. The changes of BMI and WC between the two-month and one-year visits were categorized into three groups based on estimates of normal biological variation from a reliability study by Nordhamn et al. (8) The cutoffs used were two standard deviations of intra-individual variation from their study. For BMI and WC the cutoffs were 0.6 kg/m^2^ and 5.8 cm, respectively. In the reference group the patient’s changes between the two-month and one-year visits were less than or equal to the cutoff. Patients in the other two groups had decreased and increased, respectively, their BMI and WC values more than the cutoff.

In a sensitivity analysis we examined if the analysis sample would indicate biased estimates. A Cox regression model with all covariates and BMI and WC levels at the two-month visit was estimated and compared between all available data and the analysis sample.

We confirmed the proportional-hazards assumptions of the Cox models with Schoenfeld’s test.

In Kaplan–Meier curves survival was displayed for four groups: BMI <25 kg/m^2^ and WC <102 (male)/88 (female) cm (13); BMI <25 kg/m^2^ and WC ≥102/88 cm; BMI ≥25 kg/m^2^ and WC <102/88 cm; and BMI ≥25 kg/m^2^ and WC ≥102/88 cm in which the mean of anthropometric measures from two months and one year after hospital discharge was used.

All statistical tests and confidence intervals were two-sided. Results with *P* values <0.05 were considered statistically significant without adjustments for multiplicity. The statistical analyses were performed with the statistical program package SAS version 9.4 (SAS Institute Inc., Cary, NC, USA).

## Results

**Figure 1.**
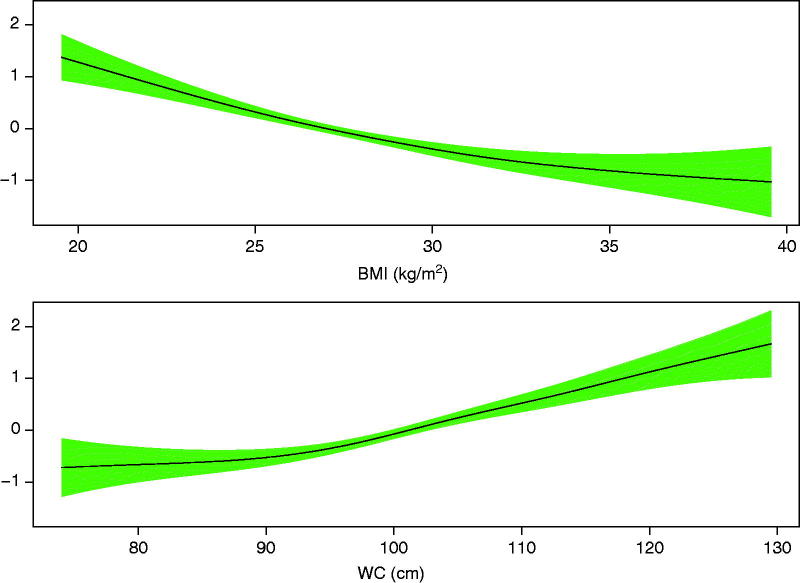
Logit of mortality risk with 95% confidence intervals versus BMI and WC, adjusted for each other.

Mean age of the 14,224 patients was 62 years (Table 1). Seventy-six per cent were men. There were 721 deaths up to 8.3 years after the one-year visit. Median follow-up time was 3.2 years. Estimated GAM models displaying associations between anthropometric variables and mortality risk showed that the associations were linear except for small deviations for high BMI values and for low WC values (Figure 1).

**Table 1.** Characteristics of the analysis sample, mean (±SD) or number (%).

Variable	*n* = 14,224
Age HIA (years)	62.0 (±8.3)
Female gender	3433 (24.1%)
Current smoker	4240 (29.8%)
Diabetes	2208 (15.5%)
Hypertension	5815 (40.9%)
Hyperlipidaemia	13,736 (96.6%)
Creatinine (µmol/L)	84.2 (±34.1)
Previous PCI	1601 (11.3%)
Previous MI	2104 (14.8%)
Previous stroke	537 (3.8%)
Previous CHF	443 (3.1%)
NSTEMI	8496 (59.7%)
PCI HIA	11,149 (78.4%)
CABG HIA	518 (3.6%)
BMI HIA (kg/m^2^)	27.5 (±4.1)
BMI 2 months (kg/m^2^)^a^	27.5 (±4.2)
BMI 12 months (kg/m^2^)^a^	27.8 (±4.3)
WC 2 months (cm)^a^	99.7 (±11.5)
WC 12 months (cm)^a^	100.4 (±11.8)

a Months after hospital discharge.

BMI = body mass index; CABG = coronary artery bypass grafting; CHF = congestive heart failure; HIA = hospital stay; MI = myocardial infarction; NSTEMI = non-ST-segment elevation myocardial infarction; PCI = percutaneous coronary intervention; STEMI = ST-segment elevation myocardial infarction; WC = waist circumference.

With adjustments for covariates and the other anthropometric measure the hazard ratio (HR) per standard deviation (SD) in a Cox proportional hazard regression model for mortality was 0.64 (95% confidence interval [CI] 0.56–0.74) for BMI and 1.55 (95% CI 1.34–1.79) for WC, and 1.43 (95% CI 1.17–1.74) for a BMI decrease from month two to one year of more than 0.6 kg/m^2^ (Table 2). No interaction effects were found between anthropometric variables and age and gender.

**Table 2.** Associations between BMI and WC, and all-cause mortality (from one year after hospital discharge), *n* = 14,224 (721 deaths).

Variable	Effect	Basic model^b^		Extended model^c^	
		HR (95% CI)	*P* value	HR (95% CI)	*P* value
BMI 2 months^a^	One SD (4.1 kg/m^2^) increase	0.62 (0.54–0.72)	<0.0001	0.64 (0.56–0.74)	<0.0001
BMI change 2 months to 1 year^a^	Decrease >0.6 kg/m^2^	1.45 (1.19–1.76)	0.0002	1.43 (1.17–1.74)	0.0004
	Change ≤0.6 kg/m^2^	1.00		1.00	
	Increase >0.6 kg/m^2^	0.96 (0.80–1.15)	0.66	0.92 (0.77–1.10)	0.36
WC 2 months^a^	One SD (11.5 cm) increase	1.84 (1.59–2.13)	<0.0001	1.55 (1.34–1.79)	<0.0001
WC change 2 months to 1 year^a^	Decrease >5.6 cm	0.80 (0.61–1.04)	0.09	0.82 (0.63–1.07)	0.15
	Change ≤5.6 cm	1.00		1.00	
	Increase >5.6 cm	1.45 (1.18–1.78)	0.0005	1.22 (0.98–1.50)	0.07

a Months after hospital discharge.

b Adjusted for the other anthropometric variable, age, and gender.

c Adjusted for the other anthropometric variable, age, gender, smoking, creatinine, diabetes, hypertension, hyperlipidaemia, previous myocardial infarction, previous stroke, previous congestive heart failure, diagnosis (STEMI/NSTEMI), percutaneous coronary intervention, coronary artery bypass grafting, and year of hospital discharge (2004–2013).

BMI = body mass index; HR = hazard ratio from Cox regression model; SD = standard deviation; WC = waist circumference.

**Figure 2.**
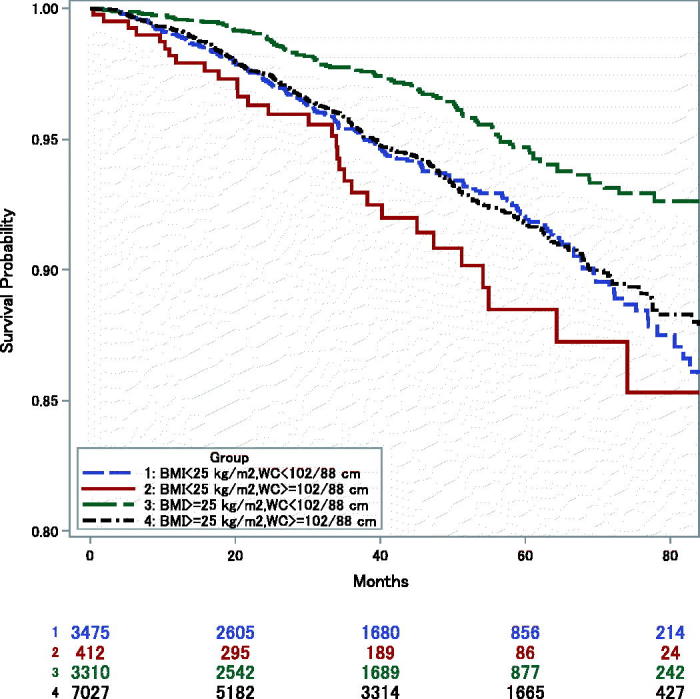
Kaplan–Meier survival estimates (from one year after hospital discharge) by anthropometric groups: Low-BMI, Low-WC = BMI<25 kg/m^2^, WC<102 (male)/88 (female) cm; Low-BMI, High-WC = BMI<25 kg/m^2^, WC≥102/88 cm; High-BMI, Low-WC = BMI≥25 kg/m^2^, WC<102/88 cm; High-BMI, High-WC = BMI≥25 kg/m^2^, WC≥102/88 cm.

Kaplan–Meier curves showed that the group with low BMI and high WC had the highest mortality, and the group with high BMI and low WC had the lowest mortality (Figure 2). The other two groups had similar mortality.

In the sensitivity analysis with a Cox regression model with all covariates and BMI and WC levels at the two-month visit was estimated with all available data (*n* = 21,214) and with the analysis sample (*n* = 14,224). HRs for BMI were 0.66 (95% CI 0.59–0.74) and 0.68 (95% CI 0.60–0.79), respectively, and HRs for WC were 1.48 (95% CI 1.32–1.66) and 1.46 (95% CI 1.27–1.67), respectively.

## Discussion

We examined a sample of 14,224 MI patients whose BMI and WC were measured two months and one year after hospital discharge. Levels of BMI and WC at two months were inversely and directly associated, respectively, with risk of all-cause mortality. Low BMI combined with high WC was associated with the highest mortality. Further, we showed that a decreased BMI, but not WC, from two months to one year was significantly associated with higher mortality risk.

To our knowledge, this study was the largest that examined the combined effects of BMI and WC on all-cause mortality for MI patients. The study was the only one that also considered fluctuations over time for these measures.

In line with our results, Coutinho et al. (9,10) showed that CAD patients with normal weight and central obesity had the highest risk of mortality. Kragelund et al. (14) showed that in patients with acute MI BMI was inversely related to mortality and that abdominal obesity appeared to be an independent predictor of mortality in men. Angerås et al. (7) examined associations between BMI and mortality in patients with acute coronary syndromes. They found that the risk for mortality decreased with increasing BMI up to 35 kg/m^2^ and then increased. Khalid et al. (15) demonstrated that patients who were overweight or obese before heart failure development had lower mortality compared with normal BMI patients.

The above-mentioned studies used BMI and WC as categorical variables. In our study we show that to a high degree of approximation the relations between BMI and WC, and mortality are linear. The implication is that decreased BMI at a given WC, or increased WC at a given BMI, is hazardous over the entire range of BMI and WC.

Central obesity, partly reflecting increased visceral fat, is coupled with the metabolic syndrome, blood lipid disorders, inflammation, insulin resistance or full-blown diabetes, and increased risk of developing cardiovascular disease (16). High BMI without central obesity may be due to muscle mass instead of increased adiposity, but could also reflect a metabolically healthy obesity, representing a phenotype with peripheral fat distribution but lower visceral fat accumulation (17). In addition, gluteofemoral body fat (subcutaneous fat in hips and legs), which is manifested in BMI, is associated with healthy metabolic profiles (18) and may thus imply lower mortality risk.

The high-risk group with central obesity and normal BMI may have received less dietary advice than overweight and obese patients. Sui et al. (19) showed that fitness is a significant mortality predictor in older adults, independent of overall or abdominal adiposity. Hence, patients with normal BMI but central obesity may have low fitness, which partly can explain the increased mortality risk for this group in our study. Furthermore, it would be interesting and clinically relevant to examine if lifestyle or drug strategies that prevent abdominal fat accumulation per se may increase survival in MI patients.

### Limitations

This prospective observational cohort study has some limitations. Residual confounding may occur. However, we were able to adjust for relevant clinical variables. Our design implies that we have not considered mortality during the first year after hospital discharge. Also, patients in the analysis sample must have performed visits at secondary prevention clinics both at two months and one year after hospital discharge. However, these restrictions entail a healthier sample than the general MI population and our risk assessments are probably underestimated. Furthermore, our findings were confirmed in a sensitivity analysis with a simple model.

The age limit of 75 years of age in the registry does not allow for conclusions to be made for patients older than this at the time of the MI. Since comorbidities potentially affecting weight may be more prevalent among the very elderly, more detailed information on existing and added comorbidities will be needed to study this group of patients.

A limitation is that no WC measurements were available from the hospital stay. Thus, we used levels from the two-month visit. This may though imply that temporal BMI and WC fluctuations in the acute disease state, due to the infarction or interventions, were avoided in the analyses.

## Conclusion

Patients with MI with normal BMI and central obesity have the highest all-cause mortality compared with other anthropometric groups. Also, a reduction of BMI implies an even higher mortality risk. BMI only is thus an inaccurate anthropometric measurement, and our results indicate that combining BMI and WC in the assessment of mortality risk in MI patients is preferable to the use of BMI alone.
